# Microbiota and Diabetes Mellitus: Role of Lipid Mediators

**DOI:** 10.3390/nu12103039

**Published:** 2020-10-03

**Authors:** Juan Salazar, Lissé Angarita, Valery Morillo, Carla Navarro, María Sofía Martínez, Maricarmen Chacín, Wheeler Torres, Arush Rajotia, Milagros Rojas, Clímaco Cano, Roberto Añez, Joselyn Rojas, Valmore Bermudez

**Affiliations:** 1 Endocrine and Metabolic Diseases Research Center, School of Medicine, University of Zulia, Maracaibo 4004, Venezuela; valerycmb@fmed.luz.edu.ve (V.M.); Cpnm18@fmed.luz.edu.ve (C.N.); mariasofia876@gmail.com (M.S.M.); wtorres@fmed.luz.edu.ve (W.T.); milagrosrojascaldera@gmail.com (M.R.); climacoc@hotmail.com (C.C.); 2 Escuela de Nutrición y Dietética, Facultad de Medicina, Universidad Andres Bello, Sede Concepción 4260000, Chile; lisse.angarita@unab.cl; 3 Facultad de Ciencias de la Salud, Universidad Simón Bolívar, Barranquilla 080001, Colombia; m.chacin@unisimonbolivar.edu.co (M.C.); v.bermudez@unisimonbolivar.edu.co (V.B.); 4 Sharma Post Graduate Institute of Medical Sciences, Rohtak, Haryana 122001, India; arush.rajotia6@gmail.com; 5 Departamento de Endocrinología y Nutrición, Hospital General Universitario Gregorio Marañón, 28009 Madrid, Spain; Roberto_anez89@hotmail.com; 6 Division of Pulmonary and Critical Care Medicine, Brigham and Women’s Hospital, Harvard Medical School, Boston, MA 02115, USA; jrojasquintero@bwh.harvard.edu

**Keywords:** diabetes, inflammation, microbiota, dysbiosis, lipopolysaccharides, short-chain fatty acids

## Abstract

Diabetes Mellitus (DM) is an inflammatory clinical entity with different mechanisms involved in its physiopathology. Among these, the dysfunction of the gut microbiota stands out. Currently, it is understood that lipid products derived from the gut microbiota are capable of interacting with cells from the immune system and have an immunomodulatory effect. In the presence of dysbiosis, the concentration of lipopolysaccharides (LPS) increases, favoring damage to the intestinal barrier. Furthermore, a pro-inflammatory environment prevails, and a state of insulin resistance and hyperglycemia is present. Conversely, during eubiosis, the production of short-chain fatty acids (SCFA) is fundamental for the maintenance of the integrity of the intestinal barrier as well as for immunogenic tolerance and appetite/satiety perception, leading to a protective effect. Additionally, it has been demonstrated that alterations or dysregulation of the gut microbiota can be reversed by modifying the eating habits of the patients or with the administration of prebiotics, probiotics, and symbiotics. Similarly, different studies have demonstrated that drugs like Metformin are capable of modifying the composition of the gut microbiota, promoting changes in the biosynthesis of LPS, and the metabolism of SCFA.

## 1. Introduction

In 2015, 1 out of 11 adult individuals suffered from Diabetes Mellitus (DM) worldwide, which estimated to 415 million cases, of which 46.5% had not been diagnosed, and mortality estimated at 5 million. Currently, it is foreseen that by the year 2040, this number may increase to 642 million, affecting 1 in every 10 adults [1]. Venezuela does not escape this reality. According to the last published data in 2011, there were 123,413 deaths associated with this disease [2]. Said numbers are alarming; therefore, prevention, early diagnosis, and treatment of diabetes Mellitus has been established as one of the priorities in patients worldwide [3,4,5].

DM is a chronic and multifactorial disease in which diverse physiopathologic mechanisms interact and lead to a persistent state of hyperglycemia [6]. In an attempt to schematize said pathways, DeFronzo proposes the “ominous octet”. This theory establishes that there are numerous mechanisms involved in the development of DM, such as beta and alpha cell dysfunction in the pancreas, a decrease in incretin effect or production, insulin resistance (IR) at the hepatic, muscular, and fatty tissue levels, increased renal glucose absorption, and the dysregulation of glucose metabolism by the central nervous system [7]. However, the “ominous eleven” are later established when Schwartz sums three additional mechanisms to this proposal: low-grade inflammation, amylin decrease, and gut microbiota modification [8].

The postulation of this last mechanism as a pathway favoring DM is based on recent evidence. This research shows that there is a dysregulation in the gut microenvironment in individuals that have type 1 diabetes (T1DM) or type 2 diabetes (T2DM) and obesity [9,10]. A critical role of gut microbiota in the regulation and functioning of the immunologic system has been discovered. This role can contribute to the development of autoimmune diseases such as T1DM. Furthermore, gut microbiota also has a role in the development of metabolic disorders such as pre-diabetes and T2DM [11,12].

In this sense, some research has demonstrated that restoration of gut microbiota can revert a significant part of metabolic alterations. Therefore, there has been a growing interest in studying the possibility of using prebiotics and probiotics in the prevention and treatment of DM [13]. Different analyses have demonstrated promising results in this aspect [11,12]. Consequently, the purpose of this narrative review is to describe the physiopathology and immunomodulatory role of gut microbiota and their lipid products in the development, prevention, and treatment of DM. For this a literature search was performed on PubMed, Excerpta Medica dataBase (EMBASE), Scopus, Web of Science, and Google Scholar databases with the terms “microbiota”, “diabetes mellitus”, “lipids” and “short-chain fatty acids”, from inception to March 2020.

## 2. Chronic Inflammation in Diabetes: Who Are the Precursors?

During the last decades, the presence of different inflammatory markers has been associated with the natural history of DM [14,15,16,17]. T1DM results from an autoimmune disorder that causes the inflammation and destruction of the pancreatic beta-cell through numerous mechanisms, leading to a deficient production of insulin [18]. T2DM is fundamentally the result of a phenomenon of IR present in the different tissues of the body, which leads to a decrease in the effects of insulin in peripheral organs [19]. Similarly, it has been stated that IR is the result of the activation of pro-inflammatory mechanisms that involve ectopic lipid accumulation, a dysfunctional adipose tissue capable of synthesizing numerous cytokines, and a dysregulated immune system, promoting a mainly inflammatory phenotype [20,21,22,23]. For this reason, DM is now considered an inflammatory clinical entity.

Also, increased levels of inflammatory mediators and markers have been observed, among which there are acute phase reactants, including C-reactive protein (CPR), interleukin-6 (IL-6), and Tumor Necrosis Factor -α (TNF-α). TNF- is one of the primary mediators that favors a pro-inflammatory state, inhibits insulin and beta-cell function, and acts in the metabolism of lipids and carbohydrates [24]. Similarly, other molecules such as interleukin-17 (IL-17), interleukin-23 (IL-23), and Transforming Growth Factor β (TGF- β) have been associated [25,26]. This pro-inflammatory state not only has a vital role in the development of the disease but also in the complications associated with it [27,28,29].

Presently, it is understood that microbial products are capable of interacting with cells from the immune system and play a vital role in the development of DM [30]. In the presence of an imbalance in the gut microorganisms, the lipopolysaccharides (LPS) derived from Gram-negative bacteria coming from the gut can generate a state of low-grade inflammation by interacting with type 4 toll-like receptors (TLR-4). These are present in the macrophages and monocytes, favoring the release of pro-inflammatory cytokines, and eventually, favoring an IR state in later stages [31,32]. Conversely, short-chain fatty acids (SCFA), in healthy individuals provide healthy energetic substrates to enterocytes, regulate the appetite/satiety process, favor an anti-inflammatory state, and activate free fatty acids type 2 and type 3 receptors (FFA2 and FFA3) and G-protein coupled receptor 109A (GPR109A), showing a protective effect [33,34].

## 3. Microbiota

There are a diverse number of microorganisms, mainly in our gut, which play a crucial role in many physiologic and metabolic processes in our body, for example, synthesis of vitamins and nutrients, regulation of metabolism, increasing bioavailability of drugs, and in protection and maturation of the immune system. These microorganisms are called “microbiota” or “gut flora” [35,36,37,38].

This “gut flora” is not static, and its composition varies with age, initial colonization by microorganisms begins at birth until three years of age [39]. It is influenced both by internal and external factors, such as the type of delivery, nutrition, and exposure to antibiotics, which leads to variability in the components of the microbiota [40,41]. Among all the gut bacteria, *Bifidobacterium* genre is the main component of the bacterial gut flora in infants [42]. Afterward, physiologically, the microbiota stays relatively stable in adults. *Bacteroidetes* and *Firmicutes* are the main components, followed by Actinobacteria and Proteobacteria [43]. Meanwhile, in older adults, there have been reports of variations because of the aging process such as dentition, decrease in chewing capacity, digestion, and intestinal transit leading to dietary changes and, therefore, changes in the composition of the gut flora [44].

The relative stability of microbiota in adults can be altered by factors such as age, nutrition, exercise, use of medication, among others, have an important influence in its composition. The process of alteration of predominant microbiota is called dysbiosis, which has been described to have associations with the development of numerous illnesses [45,46]. This alteration in microbiota can perturb the internal gut medium through diverse mechanisms, such as altering pancreatic enzyme function, biliary acid degradation, damage to the gut brush border, and by the production of dysregulated immune responses due to bacterial antigens. However, it has been proved that these alterations are reversible [47,48].

## 4. Microbiota and Diabetes: Immunomodulatory Role of Bacterial Lipid Mediators

Currently, a direct causal relationship between gut dysbiosis and the development of DM has not been identified. There are often contradictory findings; however, immunomodulatory mechanisms have been discovered, which are mediated by lipid products derived from the resident microbiota. Among these, two particular phenomena stand out in diabetic subjects: the release of LPS with pro-inflammatory effects and decrease in SCFA production [49,50].

### 4.1. Release of Lipopolysaccharides

**Figure 1:**
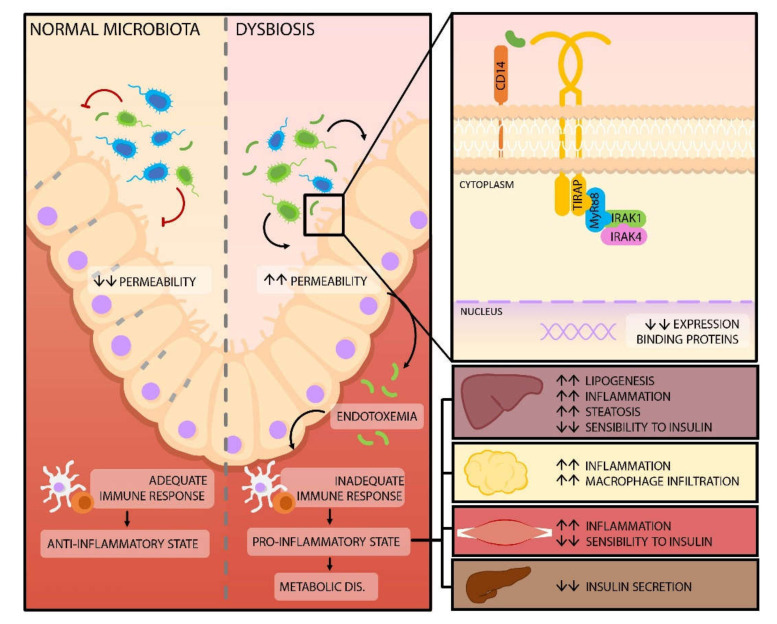
Modification of the intestinal barrier permeability. Dysbiosis, as a result of high fat intake, leads to a higher concentration of lipopolysaccharides (LPS). When LPS interacts with toll-like 4 receptors, a signaling cascade is triggered, which results in a decrease of binding proteins and, therefore, an increase in gut permeability. The resulting endotoxemia generates changes in the immune response of the host, favoring a pro-inflammatory state in different organs and tissues, which can lead to the development of metabolic diseases, such as Diabetes Mellitus. Abbreviations: CD14: Cluster of differentiation 14; TIRAP: Toll-Interleukin-1 receptor domain-containing adapter protein; MyR88: Myeloid Differentiation Gene 88; IRAK: Interleukin 1 Receptor Associated Kinase.

Gram-negative bacteria in the gut microbiota release LPS, physiologically the intestinal wall is a crucial element for controlling the transportation of these molecules to the systemic circulation, thanks to tight junctions the passage of these molecules is prevented [51]. The expression of tight junction proteins decreases with the consumption of a high-fat diet in animal models, which leads to a higher intestinal permeability and increased LPS in circulation. From there, they are transported to different tissues via serum lipoproteins (Figure 1) [51,52,53]. The mechanism through which high-fat diet decreases the expression of intestinal tight junctions involves direct and indirect pathways, which include the expression and distribution of binding protein complexes. Similarly, mechanisms such as excessive and chronic production of biliary acids capable of affecting the proteins of the intestinal barrier take place. The induction of proinflammatory molecules and cells is another mechanism that perpetuates the vicious cycle of inflammation and gut hyperpermeability as well as enhances serum LPS levels independent of permeability status. This occurs mainly through luminal micellar incorporation and stimulation of lipid raft-mediated endocytosis [54].

It has been proposed that dysbiosis induced by high-fat diets in animal models might be caused by diverse mechanisms such as the increase in biliary acid secretion and bacterial tolerance to its effects [49], as well as the rise in the concentration of H2S-producing bacteria, affecting butyrate oxidation leading to erythrocyte damage [55].

When the LPS binds to the lipopolysaccharide-binding proteins (LBP), the interaction of these molecules with a membrane protein cluster of differentiation 14 (CD14) fixed to glycosylphosphatidylinositol is facilitated, which allows for the activation of TLR-4, which triggers a signaling cascade that ends with the phosphorylation and activation of a focal adhesion kinase (FAK) in the enterocytes. Lastly, FAK regulates the activation of the primary response for myeloid differentiation gene 88 (MyD88) and kinase 4, associated with the interleukin-1 receptor (IRAK4), increasing intestinal permeability [56,57]. Similarly, there is an immune response failure with a decrease in T helper 17 lymphocytes (Th17), favoring translocation of LPS into the circulatory system [58,59].

Once in the systemic circulation, LPS act as a ligand for TLR-4, found in the membranes of cells that are part of the innate immunologic system and cells of the adipose tissue which favor the release of TNF-α and interleukins IL-1 and IL-6, among other pro-inflammatory cytokines, creating a low-grade inflammatory state capable of affecting insulin signaling and inducing insulin resistance. This step constitutes the beginning of dysfunction and cytotoxicity of the pancreatic beta-cell; both processes lead to alterations in the metabolism of lipids and carbohydrates [9,60,61,62,63].

A proposed mechanism to explain the dysfunction of the pancreatic beta-cell is based on the presence of TLR-4 in these cells. According to this theory, LPS inhibits insulin expression through the nuclear factor-kappa b (NF-ΚB) pathway, Pancreatic and Duodenal Homeobox 1 (PDX-1), and musculoaponeurotic fibrosarcoma oncogene family A (MafA) [64]. In the liver, high LPS levels induce a stress response in the endoplasmic reticulum, with subsequent production of P300 through the inositol-requiring enzyme 1 - X-box binding protein 1 (IRE1-XBP1) pathway. This protein is an acetyl-transferase capable of interrupting insulin signaling, by acting on the substrates of its receptor Insulin Receptor Substrate (IRS) 1 and 2 and decreasing its association with the beta subunit (IRβ) [65].

Several studies performed in both animals and humans have associated variations in the concentration of microorganisms that are part of the *phyla Firmicutes* (classes: Clostridia, Bacilli and Negativicutes; only this last one with Gram-negative genres) and *Bacteroidetes* (classes: Bacteroidia, Flavobacteria, Sphingobacteria, and Cytophagia; with only Gram-negative genres) to alterations in lipid and carbohydrate oxidation, respectively [66]. The high prevalence of these *phyla* (90%) in healthy subjects and their relation to different pathologies has led to using *Firmicutes/Bacteroidetes* as a marker ratio of gut microbiome dynamics [67].

In healthy subjects, the ratio favors *Bacteroidetes* over *Firmicutes* (B/F > 1); however, the *Firmicutes/Bacteroidetes* ratio undergoes an increase from birth to adulthood and it is further altered with advanced age. On the other hand, a *Firmicutes/Bacteroidetes* ratio has also been proposed for obese individuals, specifically due to the overabundance of Firmicutes. This would generate increased energy harvest, higher caloric bioavailability, and positive energy balance. All of this eventually promoting weight gain. However, the usefulness of the *Firmicutes/Bacteroidetes* ratio in obese patients continues to be controversial due to the great heterogeneity observed in the results of different series. This is likely to be caused by the number of studied subjects, the differences in methodology, and the microbiome of each population of sick individuals [68,69].

These differences in “metabolic usefulness” of the *Firmicutes/Bacteroidetes* ratio have also been seen in diabetic patients, in which an increased concentration of *Bacteroidetes* and a decrease in *Firmicutes* has been observed [70]. An increase of *Bacteroidetes* in the gut microbiota is associated with the decrease of certain bacteria such as *Akkermansia muciniphila* hampering acetogenic organisms in comparison with sulfate-reducing bacteria resulting in a lower production of butyrate, which is vital for the maintenance of gut permeability [71].

The poor sensibility of the *Firmicutes/Bacteroidetes* ratio in certain groups could be caused by the role of other bacteria in microbiome homeostasis. To this sense, the protective effect of *Bifidobacteria* has been attributed to its ability to preserve the integrity of intestinal microvilli by decreasing intestinal permeability. Likewise, they do not produce enough endotoxins to cause the stimulation of pro-inflammatory cytokines production. Furthermore, promote the production of the anti-inflammatory cytokine, maturation of dendritic cells (DCs), and T lymphocytes. They also promote an increase of T regulator lymphocyte concentration, secretion of IgA, and also have anti-oxidant functions [49,72,73,74].

Similarly, an increase in the concentration of bacteria from the *Clostridium* and *Veillonella* genre has been seen in kids with T1DM. These microorganisms ferment glucose to form propionate, acetate, and succinate from lactate. These short-chain fatty acids (SCFA) are unable to induce the synthesis of mucin, therefore, affecting binding molecules, and increasing gut permeability [75]. On the other hand, in patients with diabetes and chronic pancreatitis, a decrease in the concentration of *Faecalibacterium prausnitzii* has been observed. It is one of the greatest gut microbiota commensals, and it has anti-inflammatory properties, favoring the proliferation and growth of epithelial cells, they also promote the synthesis of binding proteins. In patients with DM, the concentration of *Ruminococcus bromii* decreases as well contributing to the production of butyrate and energy [76,77].

**Figure 2:**
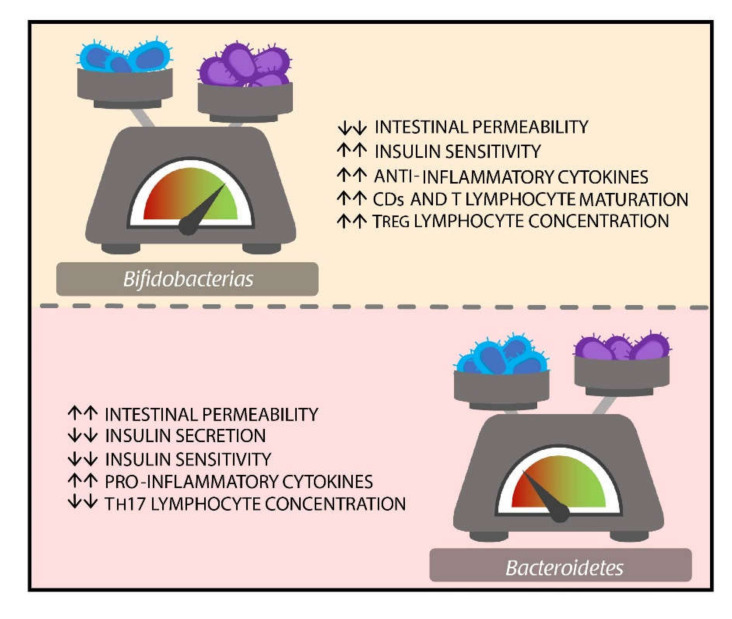
Main microorganisms seen in patients with DM2 and their potential role. DM represents a model of the wide heterogeneity of microbiome in humans and the poor role played by the *Firmicutes/Bacteroidetes* ratio as a gut microbiome homeostasis marker in certain diseases. In this particular case, the *Bifidobacterium* and *Bacteroidetes* genres are the main groups of microorganisms present in DM2 patients. Although they have potential antagonist functions, these have not been completely elucidated. Abbreviations: DM: Diabetes mellitus; Treg: Regulatory T Cells; CDs: Dendritic Cells; Th17: T Helper 17 Lymphocytes.

To this date, the main bacteria associated with DM2 in humans have been the *Bifidobacterium* and *Bacteroides* genres (Figure 2). To a lesser extent, studies have reported association with genres such as *Faecalibacterium*, *Akkermansia*, *Roseburia*, *Ruminococcus*, *Fusobacterium*, and *Blautia*; which demonstrates the heterogeneity of the gut microenvironment and the different profile of microorganisms that can exist according to its pathology [78].

### 4.2. Production of Short Chain Fatty Acids

Bacteria that comprise the gut microbiota obtain energy mainly through the fermentation of diet components such as fiber and other non-digested carbohydrates, generating SCFA as a final product [79,80]. Acetate (C2), propionate (C3), and butyrate (C4) with a molar proportion of approximately 60:20:20 respectively represent 95% of SCFA in the colon and feces in humans [81].

Among the commensal bacteria producing SCFA, the species that stand out are *Lachnospira*, *Akkermansia*, *Bifidobacterium*, *Lactobacillus*, *Ruminococcus*, *Roseburia*, *Clostridium*, *Faecalibacterium*, and *Dorea* [82]. Their adequate balance guarantees the availability of these molecules, which, besides being an essential source of energy for the colonic epithelium and liver gluconeogenesis, are capable of entering the systemic circulation and reaching peripheral tissues where they have regulatory functions in the energetic metabolism. Importantly, they play a role in the immune system, maintaining the anti/pro-inflammatory balance, which favors the integrity of the intestinal barrier, and protects against the development of DM [83]. This immunomodulatory effect of the SCFA is possible thanks to two main mechanisms, which are: the activation of cell receptors and epigenetic modifications [84], which will be discussed next.

To date, four different types of receptors that respond to SCFA, known as the free fatty acid receptors (FFAR), have been identified. These are the GPR43/FFAR2, GPR41/FFAR3, GPR109A, and Olfr78, which belong to the family of G-protein coupled receptors (GPCR) [85]. Although the three main SCFA activate FFAR2 and FFAR3, GPR109A is preferentially activated by butyrate and niacin, while Olfr78 is only activated by acetate and propionate [79]. While it is true that these receptors are expressed in a broad spectrum of tissues like the colon, small bowel, adipose tissue, skeletal muscle, liver, and pancreatic beta-cell, the immunologic effects are mainly mediated by FFAR2 and GPR109A receptors [85].

The anti-inflammatory action of SCFA like acetate and propionate takes place through the stimulation of the FFAR2 [85]. The signaling of this receptor leads to the inhibition of the nuclear translocation of the nuclear factor-kappa b (NF-κB) in different cells of the myeloid lineage leading to a decreased expression of pro-inflammatory cytokines such as TNF-α, IL-1, or IL-6 [86,87] and inducing the release of anti-inflammatory cytokines like IL-10 [88]. Moreover, different in vitro and in vivo studies have observed that the activation of this receptor is also able to modulate the recruitment of granulocytes during inflammatory bowel responses, favoring the polarization of macrophage M2 and even promoting the proliferation of Treg cells at the colonic level [85]. Furthermore, butyrate has also demonstrated anti-inflammatory effects, which are preferentially mediated by the activation of other FFAR such as GPR109A and FFAR3, inducing dendritic cells and tolerogenic macrophages, capable of preferentially generating Treg cells in the human colon [89].

Likewise, in an experimental study in mice, Chun et al., reported that the activation of FFAR2 mediated by acetate and, to a lesser degree, by propionate, increases the proliferation of innate lymphoid cells of the group 3 (ILC3) at the colonic level. Furthermore, there is also an increase in the release of IL-22 through the signaling of protein kinase B (PKB) and signal transducer and activator of transcription 2 (STAT 2), favoring the integrity of the intestinal barrier, promoting defense against pathogenic agents, and the protection against inflammation-mediated by invasive bacteria [90]. Likewise, Wu et al. reported in a preclinical study that the activation of the FFRA2 receptor mediated by acetate in dendritic cells promoted the change of class of IgA of the B cells, increasing its intestinal production and improving its protective effect [91].

The other mechanism through which SCFA, especially butyrate, have immunomodulatory effects takes place intracellularly, independently of the presence of membrane receptors. These effects occur by inhibition of class I and II histone deacetylases (HDAC), which generally promote gene expression through histone acetylation performed through in vitro human models [92,93]. This mechanism is especially important in the regulation of T lymphocytes at the systemic level since they lack the expression of SCFA receptors at physiologically relevant concentrations [79]. In this sense, SCFA promotes the differentiation of T lymphocytes in Treg cells through the inhibition of HDAC and the activation of the mTOR-S6K pathway [80]. This pathway stimulates the production of anti-inflammatory cytokines like IL-10, IL-17, and interferon-gamma (IFNγ), as well as through gene expression of Foxp3 in these cells, improving its suppressive qualities [94].

Other effects caused by SCFA inhibition of HDAC include the inhibition of neutrophil migration and the suppression of the transcription of genes involved in the response to LPS, decreasing the synthesis of pro-inflammatory mediators such as Nitric Oxide Synthase 2 (NOS2), IL-6, and IL-2 contributing to the maintenance of immune homeostasis and decrease of colonic inflammation [80].

As was previously mentioned, the key factor to observe the protective effects of SCFA is much dependent on the balance of the gut microbiota, which is determined by its composition. Therefore, in a state of eubiosis, these mechanisms can function to decrease or prevent DM [94]. The induction of tolerogenic macrophages, as well as the increase of Treg cells, can suppress the generation of autoimmune T cells, preventing the destruction of pancreatic beta-cells observed in T1DM [95]. Alternatively, the strengthening of the intestinal barrier, as well as the suppression of pro-inflammatory mediators, contributes to a decrease in the chronic inflammatory state, reducing or preventing the IR observed in T2DM [94].

It is essential to consider that in the pathophysiology of T2DM, the effects of SCFA are not limited to immunomodulatory functions. They can also intervene in the secretion of intestinal peptides that participate in the regulation of appetite and satiety, such as the glucagon-like peptide 1 (GLP-1), the YY peptide (PYY), and ghrelin [96,97]. In eubiosis conditions, propionate and butyrate interact with FFAR2 and FFAR3 receptors, stimulating the secretion of PYY and GLP-1, respectively [98]. GLP-1 participates in the regulation of insulin and glucagon secretion, gastric emptying, and food intake [94]. At the same time, PYY acts on the arcuate hypothalamic nucleus, suppressing the neuropeptide Y (NPY), which has an orexigenic effect, and stimulating POMC, which is anorexigenic, contributing to a decrease in food intake in humans [99].

**Figure 3:**
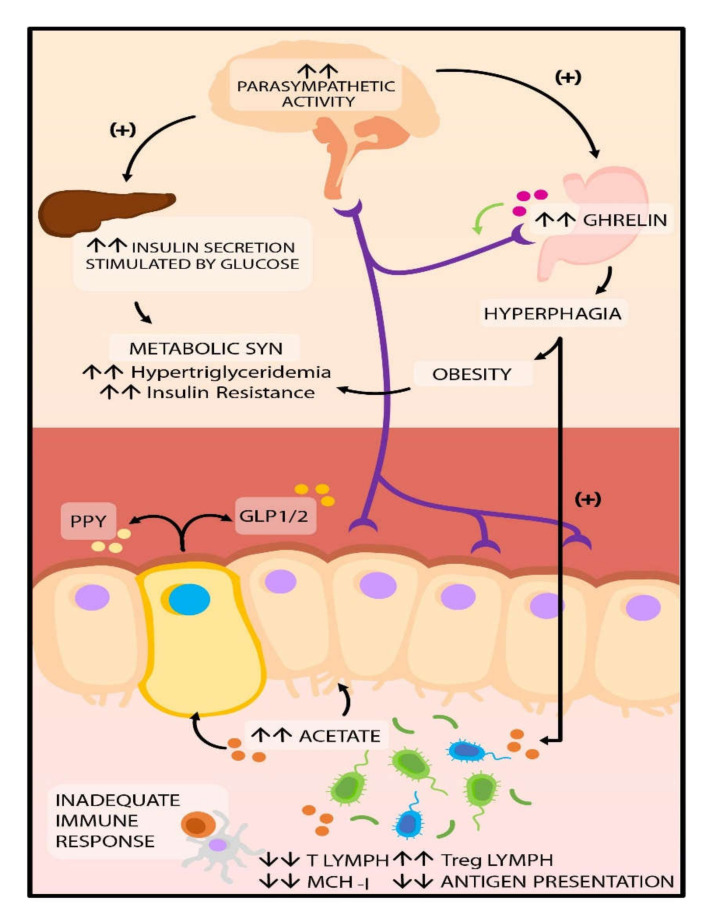
Potential regulation of hunger perception and satiety. In dysbiosis SCFA production, specifically, acetate allows the activation of the parasympathetic nervous system, which increases ghrelin secretion and, therefore, hyperphagia. It also enables the secretion of insulin, favoring the development of obesity, hyperlipidemia, and insulin resistance. Abbreviations: SCFA: short-chain fatty acids; GLP1/2: glucagon-like peptide 1/2; PPY peptide tyrosine.

However, it has been reported in preclinical studies in mice that in dysbiosis conditions caused by high-fat diets, a pathologic increase in GLP-1 takes place, which generates resistance to this peptide in the pancreatic beta-cell. This increase in GLP-1 is attributed to a decrease in the concentrations of *Lactobacillus* as well as an increase of *Bacteroides*, *Burkholderia*, and *Clostridium* [100]. Likewise, other animal models have shown that when there is gut dysbiosis, an increase in the production of SCFA acetate is capable of activating the parasympathetic nervous system, stimulating glucose-stimulated insulin secretion, increasing ghrelin secretion, and finally hyperphagia. It also affects insulin secretion, and it favors the development of obesity, hyperlipidemia, and IR [101,102] (Figure 3).

This extensive evidence that suggests a role of SCFA as critical regulators in the physiopathology of DM has mainly been observed in animal studies, which is why these findings cannot be extrapolated to humans; however, this emphasizes the need for performing controlled assays in humans. These would allow for the evaluation of therapeutic implications and establishing to which extent a dietary intervention with fiber can affect human gut microbiota and, therefore, in metabolic regulation.

## 5. Microbiota and Diabetes: Therapeutic Aspects

According to current knowledge, a significant part of alterations related to dysbiosis can be reversed by restoring gut microbiota equilibrium [103]. Based on this, the use of prebiotics (non-digestible carbohydrates), probiotics (life bacteria), and synbiotics (synergic action) have been proposed as alternative strategies for the treatment and prevention of DM (Table 1) [104,105,106,107].

Yao et al. recently published a meta-analysis that included 12 randomized and controlled clinical trials with a total population of 684 patients with T2DM. They reported that the administration of probiotics of different *Lactobacillus* and *Bifidobacterium* species was capable of significantly reducing HbA1c, fasting insulin, and Homeostasis Model Assessment of Insulin Resistance (HOMA-IR) levels and index [108]. Although the exact action mechanism of probiotics is still unknown, different authors attribute it to a decrease in plasma levels of LPS binding protein, which is a marker of endotoxemia. Furthermore, they also attribute it to a reduction in the gut inflammatory activity observed in experimental studies [109,110].

On the other hand, Alfa et al. have published a prospective, double-blind, randomized, and placebo-controlled study performed in 84 healthy patients, demonstrated that the administration of resistant starch prebiotics was able to significantly reduce insulin, glycemia, and IR levels in adults over 70 years of age [111], suggesting that this could be attributed to GLP-1 increase as well as higher fermentation of starch resistant to intestinal digestion. Alternatively, increased SCFA production, such as butyrate, due to higher fermentation of resistant starch prebiotics, has demonstrated an improvement in glucose homeostasis and insulin sensitivity through stimulation of genes involved in intestinal gluconeogenesis [112].

Other studies have focused on evaluating different dietary interventions and metabolites of the gut microbiota [123]. Such is the case of Heianza et al.; they performed a randomized study with 504 adults that were either overweight or obese. They were assigned 1 out of 4 diet plans with calorie reduction and variation in the ingestion of macronutrients for two years. The findings were that low-fat diets were able to improve the glycemic status and insulin sensitivity and were associated with a decrease of gut microbiota-dependent metabolite N-oxide of trimethylamine (TMAO) and its precursors, choline, and L-carnitine [124].

Furthermore, recent research in this area has focused on findings regarding Metformin [125] (Table 2). Metformin is the most widely employed medication for the treatment of individuals with T2DM, and it has been shown to have additional mechanisms other than activation of Activated Protein Kinase (AMPK) in metabolism regulation. It has been observed that its effects are not attenuated in mice with AMPK knockout [126]. Additionally, there is evidence that intravenous Metformin is less effective than oral Metformin [127], which suggests that the gastrointestinal tract could be an essential site for the action of this drug. Different studies have indicated that the long-term metabolic benefits of Metformin are linked to modifications in the gut microbiota, promoting changes in microbial functions, such as LPS biosynthesis, SCFA metabolism, and even biliary acid metabolism [128,129,130].

Regarding this subject, Wu et al. [131] performed a double-blind, randomized, and placebo-controlled clinical trial in patients with recent T2DM diagnosis. They had not received any prior treatment, and they were administered 1700 mg of Metformin a day (*n* = 22) or placebo (*n* = 18) for four months. The findings were that the group that received Metformin had an increase in the levels of *Akkermansia muciniphila* as well as *Bifidobacterium adolescentis*, which correlated with the improvement of HbA1c levels. They also observed that there was a significant increase in fecal levels of propionate and butyrate in the group treated with Metformin on fecal metabolomics analysis. Together, these findings could potentially contribute to the antidiabetic effect of this drug. Likewise, Napolitano et al. [126] performed a clinical trial in patients with T2DM that were treated with a stable dose of 1000 mg/day of Metformin for over three months. They reported increased GLP-1 and serum biliary acids, especially colic acid and its conjugates, which positively correlated with an abundance of bacteria from the *Firmicutes phylum* and negatively with the amount of *Bacteroides*.

Presently, the mechanism that has been best described to explain metformin-mediated beneficial effects in the metabolism has been recently proposed by Sun et al. [143], they performed a metagenomics and metabolomics serum and feces analysis in individuals that had been newly diagnosed with T2DM and had been treated with 1000 mg of Metformin twice a day for three days. The conclusion was that, independently from AMPK signaling, Metformin can inhibit the growth of *Bacteroides fragilis* by interfering in folate and methionine metabolism, decreasing the activity of bile salt hydrolase (BSH) enzyme. In consequence, there is an increase in the levels of glycoursodeoxycholic acid (GUDCA), a biliary salt capable of selectively antagonizing the farnesoid X receptor (FXR). FXR is a member of the nuclear receptor superfamily activated by a ligand, which has a critical role in the development of metabolic diseases. Therefore, there was an improvement in IR, as well as an increase in serum levels of GLP-1.

These findings are not exempt from discrepancies with other reports that have pointed out that activation of intestinal FXR can improve insulin sensitivity in mice [144,145]. Therefore, further research is necessary, as well as evaluating the possibility of creating a medication that modulates these metabolic pathways by acting only at the intestinal level, thereby decreasing systemic adverse effects.

The role of metformin in gut microbiome modulation has also been observed in non-diabetic patients. Bryrup et al. [134] analyzed the compositional changes of gut microbiome in young Danish men with no prior metformin treatment. They were part of an intervention in which they received 1 gram of metformin twice a day for six weeks. It was found that the relative abundance of genres significantly changed during the intervention, but returned to baseline levels after treatment cessation. Specifically, there was a reduced abundance of *Intestinibacter* spp. and *Clostridium* spp., as well as an increased abundance of *Escherichia*/*Shigella* spp. and *Bilophila wadsworthia*.

On the other hand, there are other oral antidiabetics such as Acarbose (Table 2), which is an inhibitor of α-glucosidase, which reduces postprandial glucose levels by diminishing intestinal absorption of glucose. There are also GLP-1 agonists, and these two drugs have also been a focus of study in this area [146,147]. In a multicentric clinical trial, Gu et al. demonstrated that Acarbose treatment is capable of modifying the composition of gut microbiota. They reported an increase in the concentration of *Lactobacillus* and *Bifidobacterium* and a decrease in *Bacteroidetes* and *Clostridium*, which modulated biliary acid metabolism. In consequence, they correlated such changes in serum concentration of biliary acids with HOMA-IR levels, lipid profile, and other clinical parameters, contributing to the antidiabetic effect of the drug [136].

Su et al. show in another clinical trial with ninety-five diabetic patients that received an intervention with Acarbose (*n* = 59) and a similar treatment without Acarbose (*n* = 36), four weeks after treatment subjects receiving Acarbose showed an increase in the concentration of *Bifidobacterium longum* and a decrease in LPS and, therefore, decreased pro-inflammatory cytokines [135].

Smith et al. proposed that Acarbose is not only able to modulate microbiota but also that by decreasing intestinal starch degradation, it increases SCFA production, especially butyrate, by intestinal microbiota [137]. Xu et al. obtained similar results when they compared gut microbiota in mice after the administration of glucosidase inhibitors. They observed that both Acarbose and Voglibose increased butyrate production. Meanwhile, the concentration of propionate and acetate only increased in the group that received Voglibose [148]. These finds suggest that the effects of Acarbose on health are partially due to the changes generated in the gut microbiota, modifying LPS and SCFA concentration as well as other products from their metabolism.

Regarding GLP-1, Wang et al. performed a clinical study to determine the differences in the gut microbiota of individuals with T2DM that were treated with Liraglutide or Metformin. They observed that those treated with Liraglutide had a significant increase in the concentration of the *Akkermansia* genre and decrease of *Suturella*, in comparison with those that received Metformin [141]. Furthermore, Zhang et al. observed that after the administration of Liraglutide to diabetic rats, there was a decrease in *Bacteroidetes/Firmicutes* ratio. Similarly, they reported a reduction in the concentration of IL-6, which could be attributed to an increase in *Lactobacilli*. The levels of these microorganisms negatively correlated with fasting glucose, a growth of SCFA-producing bacteria such as *Bacteroides acidifaciens* and *Lachnoclostridium*, which are capable of preventing low-grade inflammatory response and increasing insulin sensitivity. Furthermore, there was a decrease in the abundance of *Prevotella*, which is a bacteria that degrades mucin [149]. However, despite these findings, future research is necessary to determine how these antidiabetic medications are capable of modulating the intestinal microbiota and directing research regarding treatment to this physiopathologic target.

## 6. Conclusions

There is evidence that gut microbiota, essential for the health of the host, plays a vital role in the pathophysiology of different metabolic disorders. Among the specific mechanisms implied in the development of DM, immunomodulation mediated by lipid molecules derived from resident bacteria stands out. In dysbiosis, the increase in LPS favors damage to the intestinal barrier, as well as the presence of a pro-inflammatory environment, which promotes the development of the disease. On the contrary, in eubiosis, these contribute to the production of SCFA, which are necessary for protection against inflammation derived from pathogenic bacteria, in the maintenance of the integrity of the intestinal barrier, as well as in the development of immunogenic tolerance, preventing and decreasing the development of DM.

Preclinical and clinical studies have shown that the use of prebiotics, probiotics, synbiotics, and conventional therapeutic strategies for DM, such as dietary changes, metformin, α-glucosidase inhibitors, and more recently, GLP-1 agonist administration can modulate microbiota composition in a more effective manner when compared to alternative strategies. Due to the fact that the exact mechanism of action is unknown, a more significant number of studies are necessary to demonstrate the specific effect on the complex gut microbiota, especially in humans.

## Figures and Tables

**Table 1:** Manipulation of gut microbiota with prebiotics, probiotics, and synbiotics for diabetes treatment and prevention.

Therapeutic Strategy	Molecule/ Microorganism	Subject of Study	Effects	Ref.
Prebiotics	Chitosan oligosaccharides	Mice	↓Glycemia, IR, inflammatory mediators, lipogenesis ↑Occludins, intestinal integrity ↑ *Bacteroidetes and Akkermansia* ↓ *Firmicutes and Helicobacter*	[113]
	Oligofructose	Mice	↑Insulin and sensitivity to it ↓Lymphocyte infiltration to pancreatic islets. ↑*Bifidobacterium* ↓*Clostridium leptum*	[114]
	Inulin/Oligofructose	Humans	↓Intestinal permeability, oxidative stress, inflammation, IR, and hyperglycemia. ↑Weight loss ↑ *Bifidobacterium* and *Lactobacillus*	[104]
Probiotics	*Saccharomyces boulardii*	Mice	↓Weight and body mass; hepatic steatosis, and inflammatory state ↑ *Bacteroidetes* ↓*Firmicutes*, *Proteobacteria*, *Tenericutes*	[115]
	*Lactobacillus* *Plantarum*	Humans	Activation of TLR-2 ↑Binding proteins and protective function of the epithelium	[116]
	*Lactobacillus casei Bifidobacterium* *(alone or in combination)*	Mice	↓Fasting glucose ↓HbA1c (*B. bifidum* and in combination) ↑Blood insulin and muscle glycogen Changes to the lipid profile and antioxidant effects	[117]
	*Lactobacillus johnsonii N6.21*	Mice	↓DM incidence and oxidative stress ↑Binding proteins	[118]
	*Lactobacillus fermentum*	Mice	↓IR, blood glucose, total cholesterol, TAG, adiponectin, intestinal permeability, pro-inflammatory cytokines, and ER stress. ↑GLP-1	[119]
	*Lactobacillus rhamnosus*			
	*VSL#3* *(Bifidobacterium, Lactobacillus y Streptococcus)*	Mice	↓Weight gain, TAG and FA levels, IR and hyperinsulinemia, hepatic steatosis, and proinflammatory cytokines. Modulation of intestinal microbiota ↑Butyrate and GLP-1	[120]
Synbiotics	*Lactobacillus sporogenes* Inulin, isomalt, sorbitol y Stevia	Humans	↓insulin, glutathione, uric acid and PCR ↑HDL cholesterol	[121]
	*Lactobacillus*, *Bifidobacterium*, *Streptococcus thermophilus* Fructooligosaccharides	Humans	↓fasting glucose, HbA1c levels, and BMI	[122]

IR: Insulin Resistance; TLR-2: Toll-Like Receptors 2; HbA1c: glycated hemoglobin; DM: Diabetes Mellitus; TAG: Triglyceride; GLP-1: Glucagon-Like Peptide; ER: Endoplasmic reticulum; FA: Fatty Acids; CPR: C Reactive Protein; HDL: High Density Lipoprotein; BMI: Body Mass Index.

**Table 2:** Manipulation of gut microbiota with oral antidiabetics for diabetes treatment and prevention.

Therapeutic Strategy	Subjects of Study	Effects	Ref.
Metformin	Mice and humans	↑ Propionate and butyrate ↓ Intestinibacter spp. and *Clostridium* spp. ↑ *Escherichia*/*Shigella* spp. ↑ *Akkermansia muciniphila*	[130,132,133,134]
Acarbose	Mice and humans	↓ LPS and proinflammatory cytokines ↑ Propionate and butyrate ↓ *Clostridium* and *Bacteroides* ↑ Bifidobacterium and Lactobacillus	[135,136,137,138]
Liraglutide	Mice and humans	↑ *Bacteroidetes* ↑ *Akkermansia muciniphila* ↓ *Firmicutes* and *Proteobacteria*	[139,140,141,142]

LPS: Lipopolysaccharides.

## Abbreviations

DM	Diabetes Mellitus
LPS	Lipopolysaccharides
SCFA	Short Chain Fatty Acids
IR	Insulin Resistance
LBP	Lipopolysaccharide Binding Proteins
TLR-4	Toll-Like Receptors 4
FAK	Focal Adhesion Kinase
MyD88	Myeloid Differentiation Gene 88
IRAK4	Interleukin 1 Receptor Associated Kinase 4
Th17	T Helper 17 Lymphocytes
TNF-α	Tumor Necrosis Factor Alfa
NF-ΚB	Nuclear Factor Kappa B
PDX-1	Pancreatic and Duodenal Homeobox 1
IRS	Insulin Receptor Substrate
IRβ	Insulin Receptor Beta
DCs	Dendritic Cells
IgA	Immunoglobulin A
GLP	Glucagon-Like Peptide
GP43	Protein G43 Coupled Receptors
MCH-I	Type I Major Histocompatibility
Treg	Regulatory T Cells
TMAO	N-Oxide of Trimethylamine
AMPK	Activated Protein Kinase
BSH	Bile Salt Hydrolase Enzyme
GUDCA	Glycoursodeoxycholic Acid
FXR	Farnesoid X Receptor
CPR	C-reactive protein
IL	Interleukin
TGF-β	Transforming Growth Factor β
FFA	Free Fatty Acids Receptors
GPCR	G-protein coupled receptors
ILC3	Innate lymphoid cells of the group 3
HDAC	Histone Deacetylases
NOS2	Nitric Oxide Synthase 2
PPY	YY Peptide
NPY	Neuropeptide Y
POMC	Proopiomelanocortin

## References

[1] International Diabetes Federation (2015). IDF Diabetes Atlas. https://www.idf.org/elibrary/epidemiology-research/diabetes-atlas/13-diabetes-atlas-seventh-edition.html.

[2] Ministery of Health (2011). Bolivarian Republic of Venezuela. Anuary of Morbility. https://www.ovsalud.org/descargas/publicaciones/documentos-oficiales/Anuario-Morbilidad-2011.pdf.

[3] World Health Organization (2016). Global Report on Diabetes.

[4] De La Cruz Vargas J.A., Dos Santos F., Dyzinger W., Herzog S. (2017). Medicina Del Estilo de Vida: Trabajando Juntos Para Revertir La Epidemia de Las Enfermedades Crónicas En Latinoamérica. Cienc. Innov. Salud.

[5] Baratieri T., Dal Santo Ottoni J., Luciana Botti M., Serpa Maicel R.D.C., Gramazio Soares L. (2014). Risco Cardiovascular Em Usuários de Programa de Atenção a Hipertensos e Diabéticos Em Um Município Do Paraná-Brasil. Cienc. Innov. Salud.

[6] Morales J., Carcausto W., Varillas Y., Pérez J., Salsavilca E., Castro I., Rivera M., Quispe M. (2018). Actividad Física En Pacientes Con Diabetes Mellitus Del Primer Nivel de Atención de Lima Norte. Rev. Latinoam. Hipertens..

[7] De Fronzo R.A. (2009). From the Triumvirate to the Ominous Octet: A New Paradigm for the Treatment of Type 2 Diabetes Mellitus. Diabetes.

[8] Schwartz S.S., Epstein S., Corkey B.E., Grant S.F.A., Gavin J.R., Aguilar R.B. (2016). The Time Is Right for a New Classification System for Diabetes: Rationale and Implications of the β-Cell–Centric Classification Schema. Diabetes Care.

[9] Vatanen T., Kostic A.D., D’Hennezel E., Siljander H., Franzosa E.A., Yassour M., Kolde R., Vlamakis H., Arthur T.D., Hämäläinen A.-M. (2016). Variation in Microbiome LPS Immunogenicity Contributes to Autoimmunity in Humans. Cell.

[10] Brown K., Godovannyi A., Ma C., Zhang Y., Ahmadi-Vand Z., Dai C., Gorzelak M.A., Chan Y., Chan J.M., Lochner A. (2016). Prolonged Antibiotic Treatment Induces a Diabetogenic Intestinal Microbiome That Accelerates Diabetes in NOD Mice. ISME J..

[11] Palacios T., Vitetta L., Coulson S., Madigan C.D., Denyer G.S., Caterson I.D. (2017). The Effect of a Novel Probiotic on Metabolic Biomarkers in Adults with Prediabetes and Recently Diagnosed Type 2 Diabetes Mellitus: Study Protocol for a Randomized Controlled Trial. Trials.

[12] Peng J., Narasimhan S., Marchesi J.R., Benson A., Wong F.S., Wen L. (2014). Long Term Effect of Gut Microbiota Transfer on Diabetes Development. J. Autoimmun..

[13] Gonzalez C.M.C., Quiroz E.A.N., Lastre-Amell G., Oróstegui-Santander M.A., Peña G.E.G., Sucerquia A., Carrero L.L.S. (2020). Dislipidemia como factor de riesgo cardiovascular: Uso de probióticos en la terapéutica nutricional. Arch. Venez. Farmacol. Ter..

[14] Herder C., Færch K., Carstensen-Kirberg M., Lowe G.D., Haapakoski R., Witte D.R., Brunner E.J., Roden M., Tabák A.G., Kivimäki M. (2016). Biomarkers of Subclinical Inflammation and Increases in Glycaemia, Insulin Resistance and Beta-Cell Function in Non-Diabetic Individuals: The Whitehall II Study. Eur. J. Endocrinol..

[15] Pouvreau C., Dayre A., Butkowski E.G., de Jong B., Jelinek H.F. (2018). Inflammation and Oxidative Stress Markers in Diabetes and Hypertension. J. Inflamm. Res..

[16] Odegaard A.O., Jacobs D.R., Sanchez O.A., Goff D.C., Reiner A.P., Gross M.D. (2016). Oxidative Stress, Inflammation, Endothelial Dysfunction and Incidence of Type 2 Diabetes. Cardiovasc. Diabetol..

[17] Bermudez V., Salazar J., Gonzalez R., Ortega A., Calvo M., Olivar L.C., Morillo J., Miquilena E., Chavez-Castillo M., Chaparro R. (2019). Prevalence and Risk Factors Associated with Impaired Fasting Glucose in Adults from Maracaibo City, Venezuela. J. Diabetes Metab..

[18] Rojas J., Bermudez V., Palmar J., Martínez M.S., Olivar L.C., Nava M., Tomey D., Rojas M., Salazar J., Garicano C. (2018). Pancreatic Beta Cell Death: Novel Potential Mechanisms in Diabetes Therapy. J. Diabetes Res..

[19] Mobini R., Tremaroli V., Ståhlman M., Karlsson F., Levin M., Ljungberg M., Sohlin M., Bertéus Forslund H., Perkins R., Bäckhed F. (2017). Metabolic Effects of Lactobacillus Reuteri DSM 17938 in People with Type 2 Diabetes: A Randomized Controlled Trial. Diabetes Obes. Metab..

[20] Rodríguez Nieves R.R., Torres Ruiz L.E., Sarmiento Segarra K.B., Narea Illescas D.I., Araque Pluas I.V., Apolo Montero A.M., Ibarra Vélez L.S., Alvarado Chiquito O.L. (2019). Prevalencia de Síndrome Metabólico En Trabajadores de Una Empresa de Construcción En Guayaquil, Ecuador. Rev. Latinoam. Hipertens..

[21] Ahmad R., Thomas R., Kochumon S., Sindhu S. (2017). Increased Adipose Tissue Expression of IL-18R and Its Ligand IL-18 Associates with Inflammation and Insulin Resistance in Obesity. Immun. Inflamm. Dis..

[22] Yan Y., Li S., Liu Y., Bazzano L., He J., Mi J., Chen W. (2019). Temporal Relationship between Inflammation and Insulin Resistance and Their Joint Effect on Hyperglycemia: The Bogalusa Heart Study. Cardiovasc. Diabetol..

[23] Esser N., Legrand-Poels S., Piette J., Scheen A.J., Paquot N. (2014). Inflammation as a Link between Obesity, Metabolic Syndrome and Type 2 Diabetes. Diabetes Res. Clin. Pract..

[24] Zozulinska D., Wierusz-Wysocka B. (2006). Type 2 Diabetes Mellitus as Inflammatory Disease. Diabetes Res. Clin. Pract..

[25] Roohi A., Tabrizi M., Abbasi F., Ataie-Jafari A., Nikbin B., Larijani B., Qorbani M., Meysamie A., Asgarian-Omran H., Nikmanesh B. (2014). Serum IL-17, IL-23, and TGF-β Levels in Type 1 and Type 2 Diabetic Patients and Age-Matched Healthy Controls. Biomed. Res. Int..

[26] Abdel-Moneim A., Bakery H.H., Allam G. (2018). The Potential Pathogenic Role of IL-17/Th17 Cells in Both Type 1 and Type 2 Diabetes Mellitus. Biomed. Pharmacother..

[27] Von Scholten B.J., Reinhard H., Hansen T.W., Schalkwijk C.G., Stehouwer C., Parving H.-H., Jacobsen P.K., Rossing P. (2016). Markers of Inflammation and Endothelial Dysfunction Are Associated with Incident Cardiovascular Disease, All-Cause Mortality, and Progression of Coronary Calcification in Type 2 Diabetic Patients with Microalbuminuria. J. Diabetes Complicat..

[28] Sigurdardottir S., Zapadka T.E., Lindstrom S.I., Liu H., Taylor B.E., Lee C.A., Kern T.S., Taylor P.R. (2019). Diabetes-Mediated IL-17A Enhances Retinal Inflammation, Oxidative Stress, and Vascular Permeability. Cell. Immunol..

[29] Román-Pintos L.M., Villegas-Rivera G., Rodríguez-Carrizalez A.D., Miranda-Díaz A.G., Cardona-Muñoz E.G. (2016). Diabetic Polyneuropathy in Type 2 Diabetes Mellitus: Inflammation, Oxidative Stress, and Mitochondrial Function. J. Diabetes Res..

[30] Burcelin R. (2016). Gut Microbiota and Immune Crosstalk in Metabolic Disease. Mol. Metab..

[31] Huang X., Yan D., Xu M., Li F., Ren M., Zhang J., Wu M. (2019). Interactive Association of Lipopolysaccharide and Free Fatty Acid with the Prevalence of Type 2 Diabetes: A Community-Based Cross-Sectional Study. J. Diabetes Investig..

[32] Khondkaryan L., Margaryan S., Poghosyan D., Manukyan G. (2018). Impaired Inflammatory Response to LPS in Type 2 Diabetes Mellitus. Int. J. Inflam..

[33] Zhao L., Zhang F., Ding X., Wu G., Lam Y.Y., Wang X., Fu H., Xue X., Lu C., Ma J. (2018). Gut Bacteria Selectively Promoted by Dietary Fibers Alleviate Type 2 Diabetes. Science.

[34] Li M., van Esch B.C.A.M., Wagenaar G.T.M., Garssen J., Folkerts G., Henricks P.A.J. (2018). Pro- and Anti-Inflammatory Effects of Short Chain Fatty Acids on Immune and Endothelial Cells. Eur. J. Pharmacol..

[35] Turnbaugh P.J., Ley R.E., Hamady M., Fraser-Liggett C.M., Knight R., Gordon J.I. (2007). The Human Microbiome Project. Nature.

[36] Martí J.M., Martínez-Martínez D., Rubio T., Gracia C., Peña M., Latorre A., Moya A., Garay C.P. (2017). Health and Disease Imprinted in the Time Variability of the Human Microbiome. MSystems.

[37] Romero G. (2016). Influencia de La Microbiota Intestinal En La Enfermedad Hepática Crónica. Su Rol En El Hepatocarcinoma. Gen.

[38] Vargas-Robles D.D., Domínguez-Bello M.G. (2020). Microbiota de los indígenas del Amazonas venezolano: Influencia de los estilos de vida. Gac. Med. Caracas.

[39] Hill C.J., Lynch D.B., Murphy K., Ulaszewska M., Jeffery I.B., O’Shea C.A., Watkins C., Dempsey E., Mattivi F., Tuohy K. (2017). Evolution of Gut Microbiota Composition from Birth to 24 Weeks in the INFANTMET Cohort. Microbiome.

[40] Torres Y., Bermúdez V., Garicano C., Vilasmil N., Bautista J., Martínez M.S., Rojas-Quintero J. (2017). Desarrollo del sistema inmunológico ¿naturaleza o crianza?. Arch. Venez. Farmacol. Ter..

[41] Faneite Antique D.P., Faneite Campos J. (2020). Microbioma perinatal: Nuevos horizontes de la vida. Gac. Med. Caracas.

[42] Duranti S., Lugli G.A., Milani C., James K., Mancabelli L., Turroni F., Alessandri G., Mangifesta M., Mancino W., Ossiprandi M.C. (2019). *Bifidobacterium bifidum* and the infant gut microbiota: An intriguing case of microbe-host co-evolution. Environ. Microbiol..

[43] Biagi E., Nylund L., Candela M., Ostan R., Bucci L., Pini E., Nikkïla J., Monti D., Satokari R., Franceschi C. (2010). Through Ageing, and Beyond: Gut Microbiota and Inflammatory Status in Seniors and Centenarians. PLoS ONE.

[44] Jeffery I.B., Lynch D.B., O’Toole P.W. (2016). Composition and Temporal Stability of the Gut Microbiota in Older Persons. ISME J..

[45] Alkanani A.K., Hara N., Gottlieb P.A., Ir D., Robertson C.E., Wagner B.D., Frank D.N., Zipris D. (2015). Alterations in Intestinal Microbiota Correlate With Susceptibility to Type 1 Diabetes. Diabetes.

[46] Clarke S.F., Murphy E.F., O’Sullivan O., Lucey A.J., Humphreys M., Hogan A., Hayes P., O’Reilly M., Jeffery I.B., Wood-Martin R. (2014). Exercise and Associated Dietary Extremes Impact on Gut Microbial Diversity. Gut.

[47] Carding S., Verbeke K., Vipond D.T., Corfe B.M., Owen L.J. (2015). Dysbiosis of the Gut Microbiota in Disease. Microb. Ecol. Health Dis..

[48] Pomié C., Blasco-Baque V., Klopp P., Nicolas S., Waget A., Loubières P., Azalbert V., Puel A., Lopez F., Dray C. (2016). Triggering the Adaptive Immune System with Commensal Gut Bacteria Protects against Insulin Resistance and Dysglycemia. Mol. Metab..

[49] Morales P., Fujio S., Navarrete P., Ugalde J.A., Magne F., Carrasco-Pozo C., Tralma K., Quezada M., Hurtado C., Covarrubias N. (2016). Impact of Dietary Lipids on Colonic Function and Microbiota: An Experimental Approach Involving Orlistat-Induced Fat Malabsorption in Human Volunteers. Clin. Transl. Gastroenterol..

[50] Endesfelder D., Zu Castell W., Ardissone A., Davis-Richardson A.G., Achenbach P., Hagen M., Pflueger M., Gano K.A., Fagen J.R., Drew J.C. (2014). Compromised Gut Microbiota Networks in Children with Anti-Islet Cell Autoimmunity. Diabetes.

[51] Ghosh S.S., Wang J., Yannie P., Ghosh S. (2020). Intestinal Barrier Dysfunction, LPS Translocation, and Disease Development. J. Endocr. Soc..

[52] Cani P.D., Bibiloni R., Knauf C., Waget A., Neyrinck A.M., Delzenne N.M., Burcelin R. (2008). Changes in Gut Microbiota Control Metabolic Endotoxemia-Induced Inflammation in High-Fat Diet-Induced Obesity and Diabetes in Mice. Diabetes.

[53] Topchiy E., Cirstea M., Kong H., Boyd J., Wang Y., Russell J., Waley K. (2016). Lipopolysaccharide Is Cleared from the Circulation by Hepatocytes via the Low Density Lipoprotein Receptor. PLoS ONE.

[54] Rohr M.W., Narasimhulu C., Rudeski-Rohr T., Parthasarathy S. (2020). Negative Effects of a High-Fat Diet on Intestinal Permeability: A Review. Adv. Nutr..

[55] Lam Y.Y., Ha C.W.Y., Hoffmann J.M.A., Oscarsson J., Dinudom A., Mather T.J., Cook D.I., Hunt N.H., Caterson I.D., Holmes A.J. (2015). Effects of Dietary Fat Profile on Gut Permeability and Microbiota and Their Relationships with Metabolic Changes in Mice. Obesity.

[56] Guo S., Al-Sadi R., Said H.M., Ma T.Y. (2013). Lipopolysaccharide Causes an Increase in Intestinal Tight Junction Permeability in Vitro and in Vivo by Inducing Enterocyte Membrane Expression and Localization of TLR-4 and CD14. Am. J. Pathol..

[57] Guo S., Nighot M., Al-Sadi R., Alhmoud T., Nighot P., Ma T.Y. (2015). Lipopolysaccharide Regulation of Intestinal Tight Junction Permeability Is Mediated by TLR4 Signal Transduction Pathway Activation of FAK and MyD88. J. Immunol..

[58] Garidou L., Pomié C., Klopp P., Waget A., Charpentier J., Aloulou M., Giry A., Serino M., Stenman L., Lahtinen S. (2015). The Gut Microbiota Regulates Intestinal CD4 T Cells Expressing RORγt and Controls Metabolic Disease. Cell. Metab..

[59] Cavallari J.F., Denou E., Foley K.P., Khan W.I., Schertzer J.D. (2016). Different Th17 Immunity in Gut, Liver, and Adipose Tissues during Obesity: The Role of Diet, Genetics, and Microbes. Gut Microbes.

[60] Gomes J., de Assis J., Gonçalves R. (2017). Metabolic endotoxemia and diabetes mellitus: A systematic review. Metabolism.

[61] Matheus V.A., Monteiro L., Oliveira R.B., Maschio D.A., Collares-Buzato C.B. (2017). Butyrate Reduces High-Fat Diet-Induced Metabolic Alterations, Hepatic Steatosis and Pancreatic Beta Cell and Intestinal Barrier Dysfunctions in Prediabetic Mice. Exp. Biol. Med..

[62] Pedersen C., Gallagher E., Horton F., Ellis R.J., Ijaz U.Z., Wu H., Jaiyeola E., Diribe O., Duparc T., Cani P.D. (2016). Host–Microbiome Interactions in Human Type 2 Diabetes Following Prebiotic Fibre (Galacto-Oligosaccharide) Intake. Br. J. Nutr..

[63] Song M.J., Kim K.H., Yoon J.M., Kim J.B. (2006). Activation of Toll-like Receptor 4 Is Associated with Insulin Resistance in Adipocytes. Biochem. Biophys. Res. Commun..

[64] Amyot J., Semache M., Ferdaoussi M., Fontés G., Poitout V. (2012). Lipopolysaccharides Impair Insulin Gene Expression in Isolated Islets of Langerhans via Toll-Like Receptor-4 and NF-ΚB Signalling. PLoS ONE.

[65] Cao J., Peng J., An H., He Q., Boronina T., Guo S., White M.F., Cole P.A., He L. (2017). Endotoxemia-Mediated Activation of Acetyltransferase P300 Impairs Insulin Signaling in Obesity. Nat. Commun..

[66] Kelder T., Stroeve J.H.M., Bijlsma S., Radonjic M., Roeselers G. (2014). Correlation Network Analysis Reveals Relationships between Diet-Induced Changes in Human Gut Microbiota and Metabolic Health. Nutr. Diabetes.

[67] Mariat D., Firmesse O., Levenez F., Guimarăes V., Sokol H., Doré J., Corthier G., Furet J.P. (2009). The Firmicutes/Bacteroidetes ratio of the human microbiota changes with age. BMC Microbiol..

[68] Sikalidis A., Maykish A. (2020). The Gut Microbiome and Type 2 Diabetes Mellitus: Discussing a Complex Relationship. Biomedicines.

[69] Magne F., Gotteland M., Gauthier L., Zazueta A., Pesoa S., Navarrete P., Balamurugan R. (2020). The Firmicutes/Bacteroidetes Ratio: A Relevant Marker of Gut Dysbiosis in Obese Patients. Nutrients.

[70] Larsen N., Vogensen F., Van den Berg F., Nielsen D.S., Andreasen A.S., Pedersen B.K., Al-Soud W.A., Sørensen S.J., Hansen L.H., Jakobsen M. (2010). Gut Microbiota in Human Adults with Type 2 Diabetes Differs from Non-Diabetic Adults. PLoS ONE.

[71] Endesfelder D., Engel M., Davis-Richardson A.G., Ardissone A.N., Achenbach P., Hummel S., Winkler C., Atkinson M., Schatz D., Triplett E. (2016). Towards a Functional Hypothesis Relating Anti-Islet Cell Autoimmunity to the Dietary Impact on Microbial Communities and Butyrate Production. Microbiome.

[72] Ruiz L., Delgado S., Ruas-Madiedo P., Sánchez B., Margolles A. (2017). Bifidobacteria and Their Molecular Communication with the Immune System. Front. Microbiol..

[73] Xu J., Lian F., Zhao L., Zhao Y., Chen X., Zhang X., Guo Y., Zhang C., Zgou Q., Xue Z. (2015). Structural modulation of gut microbiota during alleviation of type 2 diabetes with a Chinese herbal formula. ISME J..

[74] Yanagibashi T., Hosono A., Oyama A., Tsuda M., Suzuki A., Hachimura S., Takahashi Y., Momose Y., Itoh K., Hirayama K. (2013). IgA Production in the Large Intestine Is Modulated by a Different Mechanism than in the Small Intestine: Bacteroides Acidifaciens Promotes IgA Production in the Large Intestine by Inducing Germinal Center Formation and Increasing the Number of IgA+ B Cells. Immunobiology.

[75] Murri M., Leiva I., Gomez-Zumaquero J.M., Tinahones F.J., Cardona F., Soriguer F., Queipo-Ortuño M.I. (2013). Gut Microbiota in Children with Type 1 Diabetes Differs from That in Healthy Children: A Case-Control Study. BMC Med..

[76] Jandhyala S.M., Madhulika A., Deepika G., Rao G.V., Reddy D.N., Subramanyam C., Sasikala M., Talukdar R. (2017). Altered Intestinal Microbiota in Patients with Chronic Pancreatitis: Implications in Diabetes and Metabolic Abnormalities. Sci. Rep..

[77] Remely M., Aumueller E., Merold C., Dworzak S., Hippe B., Zanner J., Pointner A., Brath H., Haslberger A.G. (2014). Effects of Short Chain Fatty Acid Producing Bacteria on Epigenetic Regulation of FFAR3 in Type 2 Diabetes and Obesity. Gene.

[78] Gurung M., Li Z., You H., Rodrigues R., Jump D.B., Morgun A., Shulzhenko N. (2020). Role of gut microbiota in type 2 diabetes pathophysiology. EBioMedicine.

[79] Kim C.H. (2018). Microbiota or Short-Chain Fatty Acids: Which Regulates Diabetes. Cell. Mol. Immunol..

[80] García P.O. (2020). La fibra alimentaria y su uso terapéutico en algunas enfermedades crónicas. Gac. Med. Caracas.

[81] Puddu A., Sanguineti R., Montecucco F., Viviani G.L. (2014). Evidence for the Gut Microbiota Short-Chain Fatty Acids as Key Pathophysiological Molecules Improving Diabetes. Mediat. Inflamm..

[82] Myhrstad M.C.W., Tunsjø H., Charnock C., Telle-Hansen V.H. (2020). Dietary Fiber, Gut Microbiota, and Metabolic Regulation-Current Status in Human Randomized Trials. Nutrients.

[83] Ratajczak W., Rył A., Mizerski A., Walczakiewicz K., Sipak O., Laszczyńska M. (2019). Immunomodulatory Potential of Gut Microbiome-Derived Short-Chain Fatty Acids (SCFAs). Acta Biochim. Pol..

[84] Dávila L.A., Pirela V.B., Díaz W., Villasmil N.R., León S.C., Contreras M.C.E., Bonacich K.B., Agüero S.D., Vergara P.C., Bonacich R.B., Waisundara V. (2018). The Microbiome and the Epigenetics of Diabetes Mellitus. Diabetes Food Plan.

[85] Kimura I., Ichimura A., Ohue-Kitano R., Igarashi M. (2020). Free Fatty Acid Receptors in Health and Disease. Physiol. Rev..

[86] Lee S.U., In H.J., Kwon M.S., Park B., Jo M., Kim M.-O., Cho S., Lee S., Lee H.-J., Kwak Y.S. (2013). β-Arrestin 2 Mediates G Protein-Coupled Receptor 43 Signals to Nuclear Factor-ΚB. Biol. Pharm. Bull..

[87] Cox M.A., Jackson J., Stanton M., Rojas-Triana A., Bober L., Laverty M., Yang X., Zhu F., Liu J., Wang S. (2009). Short-Chain Fatty Acids Act as Antiinflammatory Mediators by Regulating Prostaglandin E(2) and Cytokines. World J. Gastroenterol..

[88] Hernández M.A.G., Canfora E.E., Jocken J.W.E., Blaak E.E. (2019). The Short-Chain Fatty Acid Acetate in Body Weight Control and Insulin Sensitivity. Nutrients.

[89] Larasati R.A., Harbuwono D.S., Rahajeng E., Pradipta S., Nuraeni H.S., Susilowati A., Wibowo H. (2019). The Role of Butyrate on Monocyte Migration and Inflammation Response in Patient with Type 2 Diabetes Mellitus. Biomedicines.

[90] Chun E., Lavoie S., Fonseca-Pereira D., Bae S., Michaud M., Hoveyda H.R., Fraser G.L., Gallini Comeau C.A., Glickman J.N., Fuller M.H. (2019). Metabolite-Sensing Receptor Ffar2 Regulates Colonic Group 3 Innate Lymphoid Cells and Gut Immunity. Immunity.

[91] Wu W., Sun M., Chen F., Cao A.T., Liu H., Zhao Y., Huang X., Xiao Y., Yao S., Zhao Q. (2017). Microbiota Metabolite Short-Chain Fatty Acid Acetate Promotes Intestinal IgA Response to Microbiota Which Is Mediated by GPR43. Mucosal. Immunol..

[92] Kim C.H., Park J., Kim M. (2014). Gut Microbiota-Derived Short-Chain Fatty Acids, T Cells, and Inflammation. Immune Netw..

[93] Astakhova L., Ngara M., Babich O., Prosekov A., Asyakina L., Dyshlyuk L., Midtvedt T., Zhou X., Ernberg I., Matskova L. (2016). Short Chain Fatty Acids (SCFA) Reprogram Gene Expression in Human Malignant Epithelial and Lymphoid Cells. PLoS ONE.

[94] Yap Y.A., Mariño E. (2020). Dietary SCFAs Immunotherapy: Reshaping the Gut Microbiota in Diabetes. SpringerLink.

[95] Mariño E., Richards J.L., McLeod K.H., Stanley D., Yap Y.A., Knight J., McKenzie C., Kranich J., Oliveira A.C., Rossello F.J. (2017). Gut Microbial Metabolites Limit the Frequency of Autoimmune T Cells and Protect against Type 1 Diabetes. Nat. Immunol..

[96] Carpio Duran A.L., Duran Medina M.F., Andrade Valdivieso M.R., Espinoza Dunn M.A., Rodas Torres W.P., Abad Barrera L.N., Rodríguez Barzola C.V., Yagual Villon O.A. (2018). Terapia Incretinomimética: Evidencia Clínica de La Eficacia de Los Agonistas Del GLP-1R y Sus Efectos Cardio-Protectores. Rev. Latinoam. Hipertens..

[97] Rahat-Rozenbloom S., Fernandes J., Cheng J., Wolever T.M.S. (2017). Acute Increases in Serum Colonic Short-Chain Fatty Acids Elicited by Inulin Do Not Increase GLP-1 or PYY Responses but May Reduce Ghrelin in Lean and Overweight Humans. Eur. J. Clin. Nutr..

[98] Bjerg A.T., Kristensen M., Ritz C., Holst J.J., Rasmussen C., Leser T.D., Wellejus A., Astrup A. (2014). Lactobacillus Paracasei Subsp Paracasei L. Casei W8 Suppresses Energy Intake Acutely. Appetite.

[99] De Velasco P., Ferreira A., Crovesy L., Marine T., Das Graças Tavares do Carmo M., Waisundara V. (2018). Fatty Acids, Gut Microbiota, and the Genesis of Obesity. Biochemistry and Health Benefits of Fatty Acids.

[100] Grasset E., Puel A., Charpentier J., Collet X., Christensen J.E., Tercé F., Burcelin R. (2017). A Specific Gut Microbiota Dysbiosis of Type 2 Diabetic Mice Induces GLP-1 Resistance through an Enteric NO-Dependent and Gut-Brain Axis Mechanism. Cell Metab..

[101] Perry R.J., Peng L., Barry N.A., Cline G.W., Zhang D., Cardone R.L., Petersen K.F., Kibbey R.G., Goodman A.L., Shulman G.I. (2016). Acetate Mediates a Microbiome–Brain–β-Cell Axis to Promote Metabolic Syndrome. Nature.

[102] Yang M., Wang J., Wu S., Yuan L., Zhao X., Liu C., Xie J., Jia Y., Lai Y., Zhao A.Z. (2017). Duodenal GLP-1 Signaling Regulates Hepatic Glucose Production through a PKC-δ-Dependent Neurocircuitry. Cell Death Dis..

[103] Vieira A.T., Fukumori C., Ferreira C.M. (2016). New Insights into Therapeutic Strategies for Gut Microbiota Modulation in Inflammatory Diseases. Clin. Transl. Immunol..

[104] Kellow N.J., Coughlan M.T., Savige G.S., Reid C.M. (2014). Effect of Dietary Prebiotic Supplementation on Advanced Glycation, Insulin Resistance and Inflammatory Biomarkers in Adults with Pre-Diabetes: A Study Protocol for a Double-Blind Placebo-Controlled Randomised Crossover Clinical Trial. BMC Endocr. Disord..

[105] Kassaian N., Aminorroaya A., Feizi A., Jafari P., Amini M. (2017). The Effects of Probiotic and Synbiotic Supplementation on Metabolic Syndrome Indices in Adults at Risk of Type 2 Diabetes: Study Protocol for a Randomized Controlled Trial. Trials.

[106] Dávila L.A., Pirela V.B., Villasmil N.R., Cisternas S., Díaz W., Escobar M.C., Carrasco P., Durán S., Buhring K., Buhring R., Waisundara V. (2018). New Insights into Alleviating Diabetes Mellitus: Role of Gut Microbiota and a Nutrigenomic Approac. Diabetes Food Plan.

[107] Bolívar González S., Talero Barrientos E., Motilva Sánchez V. (2015). Efectos de Un Preparado Probiótico En Un Modelo de Colitis Experimental Crónica En Ratones, Inducida Por La Ingesta de Dextrano Sulfato Sódico (DSS). Cienc. Innov. Salud.

[108] Yao K., Zeng L., He Q., Wang W., Lei J., Zou X. (2017). Effect of Probiotics on Glucose and Lipid Metabolism in Type 2 Diabetes Mellitus: A Meta-Analysis of 12 Randomized Controlled Trials. Med. Sci. Monit..

[109] Naito E., Yoshida Y., Makino K., Kounoshi Y., Kunihiro S., Takahashi R., Matsuzaki T., Miyazaki K., Ishikawa F. (2011). Beneficial Effect of Oral Administration of Lactobacillus Casei Strain Shirota on Insulin Resistance in Diet-Induced Obesity Mice. J. Appl. Microbiol..

[110] Chen J.J., Wang R., Li X., Wang R. (2011). Bifidobacterium Longum Supplementation Improved High-Fat-Fed-Induced Metabolic Syndrome and Promoted Intestinal Reg I Gene Expression. Exp. Biol. Med..

[111] Alfa M.J., Strang D., Tappia P.S., Olson N., De Gagne P., Bray D., Murray B.-L., Hiebert B. (2017). A Randomized Placebo Controlled Clinical Trial to Determine the Impact of Digestion Resistant Starch MSPrebiotic^®^ on Glucose, Insulin, and Insulin Resistance in Elderly and Mid-Age Adults. Front. Med..

[112] De Vadder F., Kovatcheva-Datchary P., Goncalves D., Vinera J., Zitoun C., Duchampt A., Bäckhed F., Mithieux G. (2014). Microbiota-Generated Metabolites Promote Metabolic Benefits via Gut-Brain Neural Circuits. Cell.

[113] Zheng J., Yuan X., Cheng G., Jiao S., Feng C., Zhao X., Yin H., Du Y., Liu H. (2018). Chitosan Oligosaccharides Improve the Disturbance in Glucose Metabolism and Reverse the Dysbiosis of Gut Microbiota in Diabetic Mice. Carbohydr. Polym..

[114] Chan C., Hyslop C.M., Shrivastava V., Ochoa A., Reimer R.A., Huang C. (2016). Oligofructose as an Adjunct in Treatment of Diabetes in NOD Mice. Sci. Rep..

[115] Everard A., Matamoros S., Geurts L., Delzenne N.M., Cani P.D. (2014). Saccharomyces Boulardii Administration Changes Gut Microbiota and Reduces Hepatic Steatosis, Low-Grade Inflammation, and Fat Mass in Obese and Type 2 Diabetic Db/Db Mice. MBio.

[116] Karczewski J., Troost F.J., Konings I., Dekker J., Kleerebezem M., Brummer R.-J.M., Wells J.M. (2010). Regulation of Human Epithelial Tight Junction Proteins by Lactobacillus Plantarum in Vivo and Protective Effects on the Epithelial Barrier. Am. J. Physiol. Gastrointest. Liver Physiol..

[117] Sharma P., Bhardwaj P., Singh R. (2016). Administration of Lactobacillus Casei and Bifidobacterium Bifidum Ameliorated Hyperglycemia, Dyslipidemia, and Oxidative Stress in Diabetic Rats. Int. J. Prev. Med..

[118] Valladares R., Sankar D., Li N., Williams E., Lai K.-K., Abdelgeliel A.S., Gonzalez C.F., Wasserfall C.H., Iii J.L., Schatz D. (2010). Lactobacillus Johnsonii N6.2 Mitigates the Development of Type 1 Diabetes in BB-DP Rats. PLoS ONE.

[119] Balakumar M., Prabhu D., Sathishkumar C., Prabu P., Rokana N., Kumar R., Raghavan S., Soundarajan A., Grover S., Batish V.K. (2016). Improvement in Glucose Tolerance and Insulin Sensitivity by Probiotic Strains of Indian Gut Origin in High-Fat Diet-Fed C57BL/6J Mice. Eur. J. Nutr..

[120] Yadav H., Lee J.-H., Lloyd J., Walter P., Rane S.G. (2013). Beneficial Metabolic Effects of a Probiotic via Butyrate-Induced GLP-1 Hormone Secretion. J. Biol. Chem..

[121] Asemi Z., Khorrami-Rad A., Alizadeh S.-A., Shakeri H., Esmaillzadeh A. (2014). Effects of Synbiotic Food Consumption on Metabolic Status of Diabetic Patients: A Double-Blind Randomized Cross-over Controlled Clinical Trial. Clin. Nutr..

[122] Ebrahimi Z.S., Nasli-Esfahani E., Nadjarzade A., Mozaffari-Khosravi H. (2017). Effect of Symbiotic Supplementation on Glycemic Control, Lipid Profiles and Microalbuminuria in Patients with Non-Obese Type 2 Diabetes: A Randomized, Double-Blind, Clinical Trial. J. Diabetes Metab. Disord..

[123] Shen T.-C.D. (2017). Diet and Gut Microbiota in Health and Disease. Intestinal Microbiome: Functional Aspects in Health and Disease.

[124] Heianza Y., Sun D., Li X., DiDonato J.A., Bray G.A., Sacks F.M., Qi L. (2018). Gut Microbiota Metabolites, Amino Acid Metabolites and Improvements in Insulin Sensitivity and Glucose Metabolism: The POUNDS Lost Trial. Gut.

[125] Miranda P.J.P., Cueva C. (2019). Rol de la metformina en el tratamiento de la diabetes mellitus gestacional: Situación actual. Arch. Venez. Farmacol. Ter..

[126] Napolitano A., Miller S., Nicholls A.W., Baker D., Van Horn S., Thomas E., Rajpal D., Spivak A., Brown J.R., Nunez D.J. (2014). Novel Gut-Based Pharmacology of Metformin in Patients with Type 2 Diabetes Mellitus. PLoS ONE.

[127] Bonora E., Cigolini M., Bosello O., Zancanaro C., Capretti L., Zavaroni I., Coscelli C., Butturini U. (1984). Lack of Effect of Intravenous Metformin on Plasma Concentrations of Glucose, Insulin, C-Peptide, Glucagon and Growth Hormone in Non-Diabetic Subjects. Curr. Med. Res. Opin..

[128] Ridlon J.M., Harris S.C., Bhowmik S., Kang D.-J., Hylemon P.B. (2016). Consequences of Bile Salt Biotransformations by Intestinal Bacteria. Gut Microbes.

[129] Forslund K., Hildebrand F., Nielsen T., Falony G., Le Chatelier E., Sunagawa S., Prifti E., Vieira-Silva S., Gudmundsdottir V., Krogh Pedersen H. (2015). Disentangling Type 2 Diabetes and Metformin Treatment Signatures in the Human Gut Microbiota. Nature.

[130] De La Cuesta-Zuluaga J., Mueller N.T., Corrales-Agudelo V., Velásquez-Mejía E.P., Carmona J.A., Abad J.M., Escobar J.S. (2016). Metformin Is Associated With Higher Relative Abundance of Mucin-Degrading Akkermansia Muciniphila and Several Short-Chain Fatty Acid–Producing Microbiota in the Gut. Diabetes Care.

[131] Wu H., Esteve E., Tremaroli V., Khan M.T., Caesar R., Mannerås-Holm L., Ståhlman M., Olsson L.M., Serino M., Planas-Fèlix M. (2017). Metformin Alters the Gut Microbiome of Individuals with Treatment-Naive Type 2 Diabetes, Contributing to the Therapeutic Effects of the Drug. Nat. Med..

[132] Lee H., Ko G. (2014). Effect of Metformin on Metabolic Improvement and Gut Microbiota. Appl. Environ. Microbiol..

[133] Vallianou N., Stratigou T., Tsagarakis S. (2019). Metformin and gut microbiota: their interactions and their impact on diabetes. Hormones (Athens).

[134] Bryrup T., Thomsen C.W., Kern T., Allin K.H., Brandslund I., Jørgensen N.R., Vestergaard H., Hansen T., Hansen T.H., Pedersen O. (2019). Metformin-Induced Changes of the Gut Microbiota in Healthy Young Men: Results of a Non-Blinded, One-Armed Intervention Study. Diabetologia.

[135] Su B., Liu H., Li J., Sunli Y., Liu B., Liu D., Zhang P., Meng X. (2015). Acarbose Treatment Affects the Serum Levels of Inflammatory Cytokines and the Gut Content of Bifidobacteria in Chinese Patients with Type 2 Diabetes Mellitus. J. Diabetes.

[136] Gu Y., Wang X., Li J., Zhang Y., Zhong H., Liu R., Zhang D., Feng Q., Xie X., Hong J. (2017). Analyses of Gut Microbiota and Plasma Bile Acids Enable Stratification of Patients for Antidiabetic Treatment. Nat. Commun..

[137] Smith B.J., Miller R.A., Ericsson A.C., Harrison D.C., Strong R., Schmidt T.M. (2019). Changes in the Gut Microbiome and Fermentation Products Concurrent with Enhanced Longevity in Acarbose-Treated Mice. BMC Microbiol..

[138] Baxter N.T., Lesniak N.A., Sinani H., Schloss P.D., Koropatkin N.M. (2019). The Glucoamylase Inhibitor Acarbose Has a Diet-Dependent and Reversible Effect on the Murine Gut Microbiome. MSphere.

[139] Zhao L., Chen Y., Xia F., Abudukerimu B., Zhang W., Guo Y., Wang N., Lu Y. (2018). A Glucagon-Like Peptide-1 Receptor Agonist Lowers Weight by Modulating the Structure of Gut Microbiota. Front. Endocrinol..

[140] Wang L., Li P., Tang Z., Yan X., Feng B. (2016). Structural Modulation of the Gut Microbiota and the Relationship with Body Weight: Compared Evaluation of Liraglutide and Saxagliptin Treatment. Sci. Rep..

[141] Wang Z., Saha S., Van Horn S., Thomas E., Traini C., Sathe G., Rajpal D.K., Brown J.R. (2018). Gut Microbiome Differences between Metformin- and Liraglutide-Treated T2DM Subjects. Endocrinol. Diabetes Metab..

[142] Moreira G.V., Azevedo F.F., Ribeiro L.M., Santos A., Guadagnini D., Gama P., Liberti E.A., Saad M., Carvalho C. (2018). Liraglutide Modulates Gut Microbiota and Reduces NAFLD in Obese Mice. J. Nutr. Biochem..

[143] Sun L., Xie C., Wang G., Wu Y., Wu Q., Wang X., Liu J., Deng Y., Xia J., Chen B. (2018). Gut Microbiota and Intestinal FXR Mediate the Clinical Benefits of Metformin. Nat. Med..

[144] Fang S., Suh J.M., Reilly S.M., Yu E., Osborn O., Lackey D., Yoshihara E., Perino A., Jacinto S., Lukasheva Y. (2015). Intestinal FXR Agonism Promotes Adipose Tissue Browning and Reduces Obesity and Insulin Resistance. Nat. Med..

[145] Pathak P., Xie C., Nichols R.G., Ferrell J.M., Boehme S., Krausz K.W., Patterson A.D., Gonzalez F.J., Chiang J.Y.L. (2018). Intestine Farnesoid X Receptor Agonist and the Gut Microbiota Activate G-Protein Bile Acid Receptor-1 Signaling to Improve Metabolism. Hepatology.

[146] Zhang X., Fang Z., Zhang C., Xia H., Jie Z., Han X., Chen Y., Ji L. (2017). Effects of Acarbose on the Gut Microbiota of Prediabetic Patients: A Randomized, Double-Blind, Controlled Crossover Trial. Diabetes Ther..

[147] Remely M., Hippe B., Zanner J., Aumueller E., Brath H., Haslberger A.G. (2016). Gut Microbiota of Obese, Type 2 Diabetic Individuals Is Enriched in Faecalibacterium Prausnitzii, Akkermansia Muciniphila and Peptostreptococcus Anaerobius after Weight Loss. Endocr. Metab. Immune Disord. Drug Targets.

[148] Xu G.-D., Cai L., Ni Y.-S., Tian S.-Y., Lu Y.-Q., Wang L.-N., Chen L.-L., Ma W.-Y., Deng S.-P. (2018). Comparisons of Effects on Intestinal Short-Chain Fatty Acid Concentration after Exposure of Two Glycosidase Inhibitors in Mice. Biol. Pharm. Bull..

[149] Zhang Q., Xiao X., Zheng J., Li M., Yu M., Ping F., Wang T., Wang X. (2018). Featured Article: Structure Moderation of Gut Microbiota in Liraglutide-Treated Diabetic Male Rats. Exp. Biol. Med..

